# [(*Z*)-1-({3-[(3-Amino­prop­yl)(2-nitro­benz­yl)amino]­prop­yl}imino­meth­yl)naphthalen-2-olato]copper(II) perchlorate

**DOI:** 10.1107/S1600536811045752

**Published:** 2011-11-12

**Authors:** Reza Azadbakht, Hadi Amiri Rudbari, Saeid Menati, Giuseppe Bruno

**Affiliations:** a Department of Chemistry, Payame Noor University, Hamedan, Iran; b Dipartimento di Chimica Inorganica, Vill. S. Agata, Salita Sperone 31, Universita di Messina 98166 Messina, Italy; c Department of Chemistry, Islamic Azad University, Khorramabad Branch, Khorramabad, Iran

## Abstract

In the title compound, [Cu(C_24_H_27_N_4_O_3_)]ClO_4_, the Cu^II^ atom has a distorted square-planar coordination geometry and is surrounded by an N_3_O donor set composed of a secondary amine N, a primary amine H, an imino N and a naphthalen-2-olate O atom. An intra­molecular N—H⋯O hydrogen bond occurs. In the crystal, mol­ecules are held together by inter­molecular N—H⋯O hydrogen bonds, leading to the formation of a three-dimensional network.

## Related literature

For related structures, see: Atkins *et al.* (1993 ▶); Matsumoto *et al.* (1989 ▶); Plieger *et al.* (2004 ▶); Vigato *et al.* (2007 ▶).
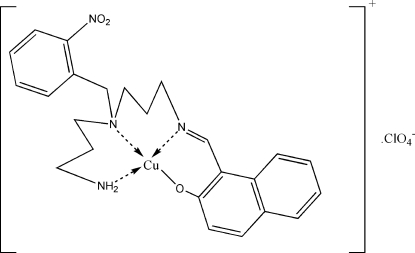


## Experimental

### 

#### Crystal data

[Cu(C_24_H_27_N_4_O_3_)]ClO_4_
*M* *_r_* = 582.50
Monoclinic, 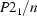

*a* = 8.1062 (4) Å
*b* = 19.2907 (8) Å
*c* = 16.0959 (7) Å
β = 102.072 (2)°
*V* = 2461.32 (19) Å^3^
*Z* = 4
Mo *K*α radiation
μ = 1.05 mm^−1^
*T* = 296 K
0.51 × 0.49 × 0.32 mm

#### Data collection

Bruker APEXII CCD diffractometer
Absorption correction: multi-scan (*SADABS*; Bruker, 2007 ▶) *T*_min_ = 0.706, *T*_max_ = 0.747
101979 measured reflections
5377 independent reflections
4605 reflections with *I* > 2σ(*I*)
*R*_int_ = 0.061

#### Refinement

*R*[*F* ^2^ > 2σ(*F* ^2^)] = 0.039
*wR*(*F* ^2^) = 0.117
*S* = 1.02
5377 reflections
342 parameters
H atoms treated by a mixture of independent and constrained refinement
Δρ_max_ = 0.59 e Å^−3^
Δρ_min_ = −0.52 e Å^−3^

### 

Data collection: *APEX2* (Bruker, 2007 ▶); cell refinement: *SAINT* (Bruker, 2007 ▶); data reduction: *SAINT*; program(s) used to solve structure: *SHELXS97* (Sheldrick, 2008 ▶); program(s) used to refine structure: *SHELXL97* (Sheldrick, 2008 ▶); molecular graphics: *XPW* (Siemens, 1996 ▶); software used to prepare material for publication: *SHELXTL* (Sheldrick, 2008 ▶).

## Supplementary Material

Crystal structure: contains datablock(s) I, global. DOI: 10.1107/S1600536811045752/rk2297sup1.cif

Structure factors: contains datablock(s) I. DOI: 10.1107/S1600536811045752/rk2297Isup2.hkl

Additional supplementary materials:  crystallographic information; 3D view; checkCIF report

## Figures and Tables

**Table 1:** Hydrogen-bond geometry (Å, °)

*D*—H⋯*A*	*D*—H	H⋯*A*	*D*⋯*A*	*D*—H⋯*A*
N1—H1⋯O3	0.82 (4)	2.46 (3)	2.948 (4)	119 (3)
N1—H2⋯O4	0.84 (3)	2.39 (4)	3.203 (5)	163 (3)
N1—H2⋯O5	0.84 (3)	2.82 (3)	3.249 (4)	114 (3)
N1—H1⋯O2^i^	0.82 (4)	2.51 (3)	3.031 (3)	122 (3)

Symmetry code: (i) 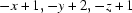.

**Fig. 1.**
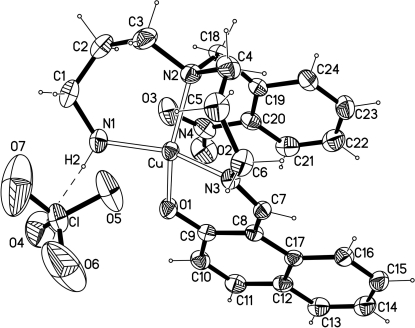
Perspective view of the title molecule with numbering of the atoms. Displacement ellipsoids are drawn at 30% probability level. The H atoms are shown as small spheres of arbitrary radius. The H2 atom is overlapped by N1 ellipsoid.

## supplementary crystallographic information

### Comment

Schiff bases have attracted increasing interest owing to their role in the understanding of molecular processes occurring in biochemistry, material science, catalysis, encapsulation, activation, transport and separation phenomena, hydrometallurgy, *etc*. (Vigato *et al.*, 2007). We report here the crystal structure of the title compound, Cu(C_24_H_27_N_4_O_3_)×ClO_4_ consists of discrete [Cu(*L*)]^+^ cations and perchlorate anions. The closest distance between Cu and O5 of ClO_4_ is 3.039 (1)Å that implies a weak coordination of oxygen to copper. The molecular structure of title compound is shown in Fig. 1. The organic ligand, *L*, coordinates in a tetradentate manner *via* three nitrogen atoms and one oxygen atom, providing a distorted square–planar arrangement about copper. The two *trans* angles at the Cu^II^ atom are about 151.48 (8)° and 158.55 (11)° and the other angles subtended at the Cu^II^ atom are close to 90°, varying from 86.12 (9)° to 99.07 (9)°. The sum of the angles subtended by the donor atoms at Cu is 369.75°.

### Experimental

A solution of NaOH (1.5 mmol) in methanol (10 cm^3^) was added to a suspension of the appropriate *N*-(2-nitrobenzyl)-*N*-(3-aminopropyl)-propane-1,3- diaminetrishydrochloride (0.5 mmol) in methanol (10 cm^3^). The mixture was stirred at room temperature for a few minutes then filtered, and the precipitate was washed well with methanol (10 cm^3^). The washings and the filtrate were combined and to this solution, copper perchlorate (0.5 mmol) and 2-hydroxy-1-naphthaldehyde (0.5 mmol) in methanol (50 cm^3^), was added. The reaction was carried out for 6 h at room temperature. The solution volume was then reduced to 10 cm^3^ by roto–evaporation and a precipitate formed on addition of a small amount of diethyl ether, which was filtered off, washed with ether, and dried under vacuo.

### Refinement

The C–based H atoms were positioned geometrically (C—H = 0.93Å and 0.97Å for CH and CH_2_ groups, respectively) and constrained to ride on their parent atoms; *U*_iso_(H) = 1.2*U*_eq_(C). The N–based H atoms were found from difference Fourier map and refined isotropically.

### Crystal data

**Table:**

[Cu(C_24_H_27_N_4_O_3_)]ClO_4_	*F*(000) = 1204
*M**_r_* = 582.50	*D*_x_ = 1.572 Mg m^−^^3^
Monoclinic, *P*2_1_/*n*	Mo *K*α radiation, λ = 0.71073 Å
Hall symbol: -P 2yn	Cell parameters from 9603 reflections
*a* = 8.1062 (4) Å	θ = 2.6–29.2°
*b* = 19.2907 (8) Å	µ = 1.05 mm^−^^1^
*c* = 16.0959 (7) Å	*T* = 296 K
β = 102.072 (2)°	Block, black
*V* = 2461.32 (19) Å^3^	0.51 × 0.49 × 0.32 mm
*Z* = 4	

### Data collection

**Table:**

Bruker APEXII CCD diffractometer	5377 independent reflections
Radiation source: fine–focus sealed tube	4605 reflections with *I* > 2σ(*I*)
graphite	*R*_int_ = 0.061
φ and ω scans	θ_max_ = 27.0°, θ_min_ = 2.5°
Absorption correction: multi-scan (*SADABS*; Bruker, 2007)	*h* = −10→10
*T*_min_ = 0.706, *T*_max_ = 0.747	*k* = −24→24
101979 measured reflections	*l* = −20→20

### Refinement

**Table:**

Refinement on *F*^2^	Primary atom site location: structure-invariant direct methods
Least-squares matrix: full	Secondary atom site location: difference Fourier map
*R*[*F*^2^ > 2σ(*F*^2^)] = 0.039	Hydrogen site location: inferred from neighbouring sites
*wR*(*F*^2^) = 0.117	H atoms treated by a mixture of independent and constrained refinement
*S* = 1.02	*w* = 1/[σ^2^(*F*_o_^2^) + (0.0736*P*)^2^ + 1.1383*P*] where *P* = (*F*_o_^2^ + 2*F*_c_^2^)/3
5377 reflections	(Δ/σ)_max_ = 0.001
342 parameters	Δρ_max_ = 0.59 e Å^−^^3^
0 restraints	Δρ_min_ = −0.52 e Å^−^^3^

### Special details

**Table:**

Geometry. All s.u.'s (except the s.u. in the dihedral angle between two l.s. planes) are estimated using the full covariance matrix. The cell s.u.'s are taken into account individually in the estimation of s.u.'s in distances, angles and torsion angles; correlations between s.u.'s in cell parameters are only used when they are defined by crystal symmetry. An approximate (isotropic) treatment of cell s.u.'s is used for estimating s.u.'s involving l.s. planes.
Refinement. Refinement of *F*^2^ against ALL reflections. The weighted *R*–factor *wR* and goodness of fit *S* are based on *F*^2^, conventional *R*–factors *R* are based on *F*, with *F* set to zero for negative *F*^2^. The threshold expression of *F*^2^ > 2σ*F*^2^ is used only for calculating *R*–factors(gt) *etc*. and is not relevant to the choice of reflections for refinement. *R*–factors based on *F*^2^ are statistically about twice as large as those based on *F*, and *R*–factors based on ALL data will be even larger.

### Fractional atomic coordinates and isotropic or equivalent isotropic displacement parameters (Å^2^)

**Table:**

	*x*	*y*	*z*	*U*_iso_*/*U*_eq_	
Cu	0.32009 (3)	0.856784 (13)	0.361933 (15)	0.03852 (11)	
N1	0.4800 (3)	0.84797 (14)	0.47232 (15)	0.0544 (5)	
C1	0.6353 (4)	0.80578 (18)	0.4829 (2)	0.0719 (8)	
H1A	0.6069	0.7573	0.4879	0.086*	
H1B	0.7120	0.8192	0.5351	0.086*	
C2	0.7232 (4)	0.81433 (19)	0.4092 (2)	0.0741 (8)	
H2A	0.7419	0.8633	0.4013	0.089*	
H2B	0.8327	0.7920	0.4236	0.089*	
C3	0.6275 (4)	0.78479 (16)	0.3269 (2)	0.0660 (7)	
H3A	0.7051	0.7797	0.2889	0.079*	
H3B	0.5894	0.7387	0.3380	0.079*	
N2	0.4785 (2)	0.82515 (10)	0.28176 (12)	0.0464 (4)	
C4	0.3820 (4)	0.78081 (14)	0.21083 (17)	0.0600 (6)	
H4A	0.3279	0.8106	0.1645	0.072*	
H4B	0.4607	0.7511	0.1897	0.072*	
C5	0.2511 (4)	0.73681 (13)	0.23789 (18)	0.0627 (7)	
H5A	0.2944	0.7203	0.2952	0.075*	
H5B	0.2288	0.6967	0.2009	0.075*	
C6	0.0886 (3)	0.77510 (13)	0.23556 (17)	0.0546 (6)	
H6A	0.0303	0.7812	0.1769	0.065*	
H6B	0.0167	0.7477	0.2640	0.065*	
N3	0.1174 (2)	0.84326 (9)	0.27678 (11)	0.0406 (4)	
C7	0.0059 (3)	0.89113 (11)	0.25349 (13)	0.0399 (4)	
H7	−0.0785	0.8818	0.2061	0.048*	
C8	−0.0007 (3)	0.95679 (11)	0.29327 (13)	0.0390 (4)	
C17	−0.1169 (3)	1.00936 (11)	0.25241 (14)	0.0407 (4)	
C16	−0.2083 (3)	1.00439 (14)	0.16799 (15)	0.0498 (5)	
H16	−0.1943	0.9656	0.1358	0.060*	
C15	−0.3186 (3)	1.05619 (15)	0.13210 (17)	0.0569 (6)	
H15	−0.3781	1.0516	0.0764	0.068*	
C14	−0.3413 (3)	1.11485 (15)	0.1783 (2)	0.0608 (7)	
H14	−0.4162	1.1492	0.1537	0.073*	
C13	−0.2539 (3)	1.12185 (14)	0.25950 (19)	0.0559 (6)	
H13	−0.2695	1.1613	0.2902	0.067*	
C12	−0.1396 (3)	1.07032 (12)	0.29802 (16)	0.0465 (5)	
C11	−0.0439 (3)	1.07871 (13)	0.38245 (17)	0.0535 (6)	
H11	−0.0606	1.1180	0.4131	0.064*	
C10	0.0696 (3)	1.03129 (13)	0.41882 (15)	0.0514 (5)	
H10	0.1313	1.0390	0.4736	0.062*	
C9	0.0979 (3)	0.96920 (12)	0.37529 (14)	0.0422 (5)	
O1	0.2131 (2)	0.92738 (9)	0.41511 (10)	0.0530 (4)	
C18	0.5462 (3)	0.88515 (14)	0.23958 (15)	0.0494 (5)	
H18A	0.5944	0.8674	0.1935	0.059*	
H18B	0.6367	0.9066	0.2805	0.059*	
C19	0.4182 (3)	0.94026 (13)	0.20450 (14)	0.0457 (5)	
C24	0.3379 (4)	0.93601 (15)	0.11856 (16)	0.0585 (6)	
H24	0.3629	0.8988	0.0866	0.070*	
C23	0.2235 (4)	0.98488 (18)	0.07963 (18)	0.0684 (8)	
H23	0.1709	0.9796	0.0228	0.082*	
C22	0.1872 (4)	1.04122 (17)	0.1245 (2)	0.0702 (8)	
H22	0.1095	1.0741	0.0985	0.084*	
C21	0.2654 (4)	1.04867 (14)	0.2073 (2)	0.0623 (7)	
H21	0.2421	1.0871	0.2378	0.075*	
C20	0.3800 (3)	0.99919 (12)	0.24637 (15)	0.0479 (5)	
N4	0.4641 (3)	1.01508 (12)	0.33436 (14)	0.0576 (5)	
O3	0.5511 (3)	0.97071 (12)	0.37640 (13)	0.0770 (6)	
O2	0.4446 (4)	1.07214 (12)	0.36190 (16)	0.0890 (7)	
Cl	0.19969 (9)	0.70592 (4)	0.51751 (4)	0.06081 (18)	
O4	0.2496 (4)	0.76098 (17)	0.57398 (17)	0.1104 (10)	
O5	0.2004 (5)	0.72505 (17)	0.43448 (15)	0.1153 (11)	
O6	0.0406 (7)	0.6830 (4)	0.5193 (3)	0.240 (4)	
O7	0.3086 (9)	0.6533 (2)	0.5438 (3)	0.216 (3)	
H1	0.517 (4)	0.8868 (19)	0.487 (2)	0.068 (10)*	
H2	0.419 (4)	0.8333 (18)	0.504 (2)	0.064 (9)*	

### Atomic displacement parameters (Å^2^)

**Table:**

	*U*^11^	*U*^22^	*U*^33^	*U*^12^	*U*^13^	*U*^23^
Cu	0.04088 (16)	0.03732 (16)	0.03504 (15)	0.00157 (9)	0.00268 (11)	−0.00284 (9)
N1	0.0597 (13)	0.0547 (13)	0.0434 (11)	0.0018 (10)	−0.0021 (10)	0.0004 (10)
C1	0.0653 (17)	0.0725 (19)	0.0671 (17)	0.0133 (14)	−0.0109 (14)	0.0159 (14)
C2	0.0511 (15)	0.085 (2)	0.080 (2)	0.0195 (14)	−0.0004 (14)	0.0092 (17)
C3	0.0604 (16)	0.0568 (15)	0.0821 (19)	0.0214 (13)	0.0182 (14)	0.0061 (14)
N2	0.0489 (10)	0.0414 (10)	0.0487 (10)	0.0063 (8)	0.0097 (8)	−0.0082 (8)
C4	0.0767 (17)	0.0516 (14)	0.0525 (14)	0.0014 (12)	0.0156 (12)	−0.0157 (11)
C5	0.0880 (19)	0.0361 (12)	0.0606 (15)	−0.0022 (12)	0.0082 (14)	−0.0118 (11)
C6	0.0595 (14)	0.0393 (12)	0.0583 (14)	−0.0091 (10)	−0.0028 (11)	−0.0071 (10)
N3	0.0423 (9)	0.0380 (9)	0.0398 (9)	−0.0053 (7)	0.0051 (7)	−0.0034 (7)
C7	0.0350 (10)	0.0452 (11)	0.0384 (10)	−0.0032 (8)	0.0054 (8)	0.0007 (8)
C8	0.0372 (10)	0.0418 (11)	0.0399 (10)	−0.0015 (8)	0.0124 (8)	0.0008 (8)
C17	0.0364 (10)	0.0428 (11)	0.0456 (11)	−0.0014 (8)	0.0147 (8)	0.0067 (9)
C16	0.0475 (12)	0.0529 (13)	0.0498 (12)	0.0025 (10)	0.0120 (10)	0.0054 (10)
C15	0.0500 (13)	0.0642 (16)	0.0556 (14)	0.0036 (11)	0.0090 (11)	0.0163 (12)
C14	0.0560 (14)	0.0513 (14)	0.0769 (18)	0.0111 (11)	0.0180 (13)	0.0212 (13)
C13	0.0568 (14)	0.0421 (12)	0.0725 (17)	0.0059 (11)	0.0221 (13)	0.0081 (11)
C12	0.0455 (11)	0.0411 (11)	0.0572 (13)	0.0003 (9)	0.0205 (10)	0.0062 (10)
C11	0.0610 (14)	0.0434 (12)	0.0595 (14)	0.0028 (10)	0.0206 (11)	−0.0075 (10)
C10	0.0573 (13)	0.0536 (14)	0.0444 (12)	0.0003 (11)	0.0132 (10)	−0.0089 (10)
C9	0.0416 (11)	0.0454 (11)	0.0417 (11)	0.0015 (9)	0.0131 (8)	−0.0012 (9)
O1	0.0584 (10)	0.0587 (10)	0.0380 (8)	0.0146 (8)	0.0013 (7)	−0.0091 (7)
C18	0.0466 (12)	0.0594 (14)	0.0459 (12)	0.0032 (10)	0.0183 (10)	−0.0025 (10)
C19	0.0483 (12)	0.0503 (12)	0.0419 (11)	−0.0017 (10)	0.0171 (9)	0.0020 (9)
C24	0.0666 (16)	0.0669 (16)	0.0443 (12)	0.0028 (13)	0.0168 (11)	0.0004 (11)
C23	0.0700 (17)	0.083 (2)	0.0499 (14)	0.0015 (15)	0.0079 (13)	0.0171 (14)
C22	0.0640 (17)	0.0654 (18)	0.083 (2)	0.0069 (13)	0.0186 (15)	0.0270 (16)
C21	0.0673 (16)	0.0453 (13)	0.0804 (19)	0.0004 (12)	0.0294 (14)	0.0091 (13)
C20	0.0533 (12)	0.0439 (12)	0.0516 (12)	−0.0081 (10)	0.0221 (10)	0.0020 (10)
N4	0.0713 (14)	0.0498 (12)	0.0566 (12)	−0.0154 (10)	0.0244 (11)	−0.0069 (10)
O3	0.1088 (17)	0.0651 (13)	0.0520 (11)	−0.0091 (12)	0.0052 (11)	−0.0036 (10)
O2	0.122 (2)	0.0594 (13)	0.0867 (16)	−0.0078 (13)	0.0250 (14)	−0.0305 (12)
Cl	0.0796 (4)	0.0619 (4)	0.0372 (3)	−0.0102 (3)	0.0036 (3)	0.0037 (2)
O4	0.142 (3)	0.103 (2)	0.0761 (16)	0.0126 (18)	−0.0001 (16)	−0.0345 (15)
O5	0.172 (3)	0.122 (2)	0.0487 (12)	−0.050 (2)	0.0170 (15)	0.0102 (13)
O6	0.198 (5)	0.412 (9)	0.109 (3)	−0.198 (6)	0.029 (3)	0.016 (4)
O7	0.366 (8)	0.121 (3)	0.116 (3)	0.115 (4)	−0.054 (4)	−0.030 (2)

### Geometric parameters (Å, °)

**Table:**

Cu—O1	1.9104 (16)	C16—C15	1.384 (3)
Cu—N3	1.9245 (18)	C16—H16	0.9300
Cu—N1	1.976 (2)	C15—C14	1.387 (4)
Cu—N2	2.0925 (19)	C15—H15	0.9300
N1—C1	1.479 (4)	C14—C13	1.357 (4)
N1—H1	0.82 (4)	C14—H14	0.9300
N1—H2	0.84 (3)	C13—C12	1.411 (3)
C1—C2	1.515 (5)	C13—H13	0.9300
C1—H1A	0.9700	C12—C11	1.426 (4)
C1—H1B	0.9700	C11—C10	1.342 (4)
C2—C3	1.500 (5)	C11—H11	0.9300
C2—H2A	0.9700	C10—C9	1.430 (3)
C2—H2B	0.9700	C10—H10	0.9300
C3—N2	1.492 (3)	C9—O1	1.297 (3)
C3—H3A	0.9700	C18—C19	1.510 (3)
C3—H3B	0.9700	C18—H18A	0.9700
N2—C18	1.503 (3)	C18—H18B	0.9700
N2—C4	1.508 (3)	C19—C20	1.389 (3)
C4—C5	1.493 (4)	C19—C24	1.402 (3)
C4—H4A	0.9700	C24—C23	1.378 (4)
C4—H4B	0.9700	C24—H24	0.9300
C5—C6	1.504 (4)	C23—C22	1.371 (5)
C5—H5A	0.9700	C23—H23	0.9300
C5—H5B	0.9700	C22—C21	1.358 (4)
C6—N3	1.469 (3)	C22—H22	0.9300
C6—H6A	0.9700	C21—C20	1.388 (4)
C6—H6B	0.9700	C21—H21	0.9300
N3—C7	1.292 (3)	C20—N4	1.470 (3)
C7—C8	1.425 (3)	N4—O2	1.209 (3)
C7—H7	0.9300	N4—O3	1.220 (3)
C8—C9	1.413 (3)	Cl—O7	1.354 (4)
C8—C17	1.445 (3)	Cl—O6	1.369 (4)
C17—C16	1.408 (3)	Cl—O5	1.388 (2)
C17—C12	1.419 (3)	Cl—O4	1.402 (3)
			
O1—Cu—N3	90.91 (7)	C7—C8—C17	120.07 (19)
O1—Cu—N1	86.12 (9)	C16—C17—C12	117.2 (2)
N3—Cu—N1	158.55 (11)	C16—C17—C8	123.6 (2)
O1—Cu—N2	151.48 (8)	C12—C17—C8	119.3 (2)
N3—Cu—N2	93.65 (8)	C15—C16—C17	121.1 (2)
N1—Cu—N2	99.07 (9)	C15—C16—H16	119.4
C1—N1—Cu	122.3 (2)	C17—C16—H16	119.4
C1—N1—H1	103 (2)	C16—C15—C14	120.8 (2)
Cu—N1—H1	108 (2)	C16—C15—H15	119.6
C1—N1—H2	110 (2)	C14—C15—H15	119.6
Cu—N1—H2	103 (2)	C13—C14—C15	119.7 (2)
H1—N1—H2	111 (3)	C13—C14—H14	120.1
N1—C1—C2	112.2 (2)	C15—C14—H14	120.1
N1—C1—H1A	109.2	C14—C13—C12	121.1 (3)
C2—C1—H1A	109.2	C14—C13—H13	119.5
N1—C1—H1B	109.2	C12—C13—H13	119.5
C2—C1—H1B	109.2	C13—C12—C17	120.1 (2)
H1A—C1—H1B	107.9	C13—C12—C11	121.0 (2)
C3—C2—C1	114.1 (3)	C17—C12—C11	118.9 (2)
C3—C2—H2A	108.7	C10—C11—C12	121.7 (2)
C1—C2—H2A	108.7	C10—C11—H11	119.2
C3—C2—H2B	108.7	C12—C11—H11	119.2
C1—C2—H2B	108.7	C11—C10—C9	121.5 (2)
H2A—C2—H2B	107.6	C11—C10—H10	119.2
N2—C3—C2	116.6 (2)	C9—C10—H10	119.2
N2—C3—H3A	108.1	O1—C9—C8	124.3 (2)
C2—C3—H3A	108.1	O1—C9—C10	116.8 (2)
N2—C3—H3B	108.1	C8—C9—C10	118.8 (2)
C2—C3—H3B	108.1	C9—O1—Cu	124.70 (14)
H3A—C3—H3B	107.3	N2—C18—C19	115.07 (18)
C3—N2—C18	106.7 (2)	N2—C18—H18A	108.5
C3—N2—C4	108.2 (2)	C19—C18—H18A	108.5
C18—N2—C4	105.99 (19)	N2—C18—H18B	108.5
C3—N2—Cu	112.97 (17)	C19—C18—H18B	108.5
C18—N2—Cu	112.54 (13)	H18A—C18—H18B	107.5
C4—N2—Cu	110.16 (16)	C20—C19—C24	115.0 (2)
C5—C4—N2	112.9 (2)	C20—C19—C18	126.9 (2)
C5—C4—H4A	109.0	C24—C19—C18	117.9 (2)
N2—C4—H4A	109.0	C23—C24—C19	122.5 (3)
C5—C4—H4B	109.0	C23—C24—H24	118.7
N2—C4—H4B	109.0	C19—C24—H24	118.7
H4A—C4—H4B	107.8	C22—C23—C24	120.1 (3)
C4—C5—C6	112.7 (2)	C22—C23—H23	120.0
C4—C5—H5A	109.1	C24—C23—H23	120.0
C6—C5—H5A	109.1	C21—C22—C23	119.6 (3)
C4—C5—H5B	109.1	C21—C22—H22	120.2
C6—C5—H5B	109.1	C23—C22—H22	120.2
H5A—C5—H5B	107.8	C22—C21—C20	120.2 (3)
N3—C6—C5	111.9 (2)	C22—C21—H21	119.9
N3—C6—H6A	109.2	C20—C21—H21	119.9
C5—C6—H6A	109.2	C21—C20—C19	122.6 (2)
N3—C6—H6B	109.2	C21—C20—N4	115.2 (2)
C5—C6—H6B	109.2	C19—C20—N4	122.2 (2)
H6A—C6—H6B	107.9	O2—N4—O3	122.7 (3)
C7—N3—C6	118.23 (19)	O2—N4—C20	118.3 (3)
C7—N3—Cu	123.45 (15)	O3—N4—C20	119.1 (2)
C6—N3—Cu	118.33 (15)	O7—Cl—O6	107.8 (5)
N3—C7—C8	126.47 (19)	O7—Cl—O5	111.5 (3)
N3—C7—H7	116.8	O6—Cl—O5	107.4 (2)
C8—C7—H7	116.8	O7—Cl—O4	106.5 (2)
C9—C8—C7	120.22 (19)	O6—Cl—O4	112.2 (3)
C9—C8—C17	119.6 (2)	O5—Cl—O4	111.4 (2)
			
O1—Cu—N1—C1	−170.5 (3)	C16—C15—C14—C13	0.4 (4)
N3—Cu—N1—C1	106.9 (3)	C15—C14—C13—C12	0.0 (4)
N2—Cu—N1—C1	−18.7 (3)	C14—C13—C12—C17	−1.2 (4)
Cu—N1—C1—C2	41.3 (4)	C14—C13—C12—C11	178.1 (2)
N1—C1—C2—C3	−67.9 (4)	C16—C17—C12—C13	1.9 (3)
C1—C2—C3—N2	75.1 (4)	C8—C17—C12—C13	−179.3 (2)
C2—C3—N2—C18	77.0 (3)	C16—C17—C12—C11	−177.4 (2)
C2—C3—N2—C4	−169.4 (3)	C8—C17—C12—C11	1.4 (3)
C2—C3—N2—Cu	−47.2 (3)	C13—C12—C11—C10	−177.6 (2)
O1—Cu—N2—C3	118.2 (2)	C17—C12—C11—C10	1.7 (4)
N3—Cu—N2—C3	−143.12 (18)	C12—C11—C10—C9	−1.3 (4)
N1—Cu—N2—C3	19.6 (2)	C7—C8—C9—O1	8.8 (3)
O1—Cu—N2—C18	−2.7 (2)	C17—C8—C9—O1	−175.4 (2)
N3—Cu—N2—C18	96.00 (16)	C7—C8—C9—C10	−170.5 (2)
N1—Cu—N2—C18	−101.32 (17)	C17—C8—C9—C10	5.2 (3)
O1—Cu—N2—C4	−120.71 (19)	C11—C10—C9—O1	178.4 (2)
N3—Cu—N2—C4	−22.04 (17)	C11—C10—C9—C8	−2.2 (4)
N1—Cu—N2—C4	140.64 (18)	C8—C9—O1—Cu	19.6 (3)
C3—N2—C4—C5	89.5 (3)	C10—C9—O1—Cu	−161.05 (17)
C18—N2—C4—C5	−156.4 (2)	N3—Cu—O1—C9	−32.2 (2)
Cu—N2—C4—C5	−34.4 (3)	N1—Cu—O1—C9	169.1 (2)
N2—C4—C5—C6	83.4 (3)	N2—Cu—O1—C9	67.2 (3)
C4—C5—C6—N3	−47.7 (3)	C3—N2—C18—C19	−168.7 (2)
C5—C6—N3—C7	154.6 (2)	C4—N2—C18—C19	76.2 (2)
C5—C6—N3—Cu	−25.0 (3)	Cu—N2—C18—C19	−44.3 (2)
O1—Cu—N3—C7	26.90 (18)	N2—C18—C19—C20	91.4 (3)
N1—Cu—N3—C7	108.6 (3)	N2—C18—C19—C24	−94.6 (3)
N2—Cu—N3—C7	−124.94 (18)	C20—C19—C24—C23	−3.0 (4)
O1—Cu—N3—C6	−153.60 (18)	C18—C19—C24—C23	−177.8 (3)
N1—Cu—N3—C6	−71.9 (3)	C19—C24—C23—C22	1.6 (5)
N2—Cu—N3—C6	54.57 (18)	C24—C23—C22—C21	0.4 (5)
C6—N3—C7—C8	170.9 (2)	C23—C22—C21—C20	−0.7 (4)
Cu—N3—C7—C8	−9.6 (3)	C22—C21—C20—C19	−1.0 (4)
N3—C7—C8—C9	−14.2 (3)	C22—C21—C20—N4	176.4 (2)
N3—C7—C8—C17	170.1 (2)	C24—C19—C20—C21	2.7 (3)
C9—C8—C17—C16	173.8 (2)	C18—C19—C20—C21	176.9 (2)
C7—C8—C17—C16	−10.4 (3)	C24—C19—C20—N4	−174.5 (2)
C9—C8—C17—C12	−4.9 (3)	C18—C19—C20—N4	−0.3 (4)
C7—C8—C17—C12	170.84 (19)	C21—C20—N4—O2	−9.1 (3)
C12—C17—C16—C15	−1.5 (3)	C19—C20—N4—O2	168.3 (2)
C8—C17—C16—C15	179.7 (2)	C21—C20—N4—O3	171.4 (2)
C17—C16—C15—C14	0.4 (4)	C19—C20—N4—O3	−11.2 (3)

### Hydrogen-bond geometry (Å, °)

**Table:**

*D*—H···*A*	*D*—H	H···*A*	*D*···*A*	*D*—H···*A*
N1—H1···O3	0.82 (4)	2.46 (3)	2.948 (4)	119 (3)
N1—H2···O4	0.84 (3)	2.39 (4)	3.203 (5)	163 (3)
N1—H2···O5	0.84 (3)	2.82 (3)	3.249 (4)	114 (3)
N1—H1···O2^i^	0.82 (4)	2.51 (3)	3.031 (3)	122 (3)

Symmetry codes: (i) −*x*+1, −*y*+2, −*z*+1.
